# Mitochondrial CPT1A: Insights into structure, function, and basis for drug development

**DOI:** 10.3389/fphar.2023.1160440

**Published:** 2023-03-23

**Authors:** Kai Liang

**Affiliations:** School of Life Science, Peking University, Beijing, China

**Keywords:** CPT1a, FATTY ACID β-OXIDATION, cancer, inhibitor, drug development

## Abstract

Carnitine Palmitoyl-Transferase1A (CPT1A) is the rate-limiting enzyme in the fatty acid β-oxidation, and its deficiency or abnormal regulation can result in diseases like metabolic disorders and various cancers. Therefore, CPT1A is a desirable drug target for clinical therapy. The deep comprehension of human CPT1A is crucial for developing the therapeutic inhibitors like Etomoxir. CPT1A is an appealing druggable target for cancer therapies since it is essential for the survival, proliferation, and drug resistance of cancer cells. It will help to lower the risk of cancer recurrence and metastasis, reduce mortality, and offer prospective therapy options for clinical treatment if the effects of CPT1A on the lipid metabolism of cancer cells are inhibited. Targeted inhibition of CPT1A can be developed as an effective treatment strategy for cancers from a metabolic perspective. However, the pathogenic mechanism and recent progress of CPT1A in diseases have not been systematically summarized. Here we discuss the functions of CPT1A in health and diseases, and prospective therapies targeting CPT1A. This review summarizes the current knowledge of CPT1A, hoping to prompt further understanding of it, and provide foundation for CPT1A-targeting drug development.

## Introduction

Globally, the prevalence of obesity has increased, and more and more attention has been paid to lipid metabolism (Bluher, 2019). Fatty acid oxidation (FAO), a process that occurs within cells, is an important energy source. Diabetes patients cannot use sugar as their primary source of energy since they have poor insulin sensitivity (Shepherd and Kahn, 1999). Additionally, the majority of cancer cells use lipid metabolism as a source of energy (Munir et al., 2019). Reducing or inhibiting FAO can turn off the energy source of cancer cells and starve them to death (Bergers and Fendt, 2021).

The majority of FAO happens in mitochondrion. Long chain fatty acids (LCFAs) cannot directly enter the mitochondrial inner membrane, and CPT1A is required to convert acyl-CoA (carbon chain longer than 12) into acyl-carnitine (Ceccarelli et al., 2011). CACT (carnitine/acylcarnitine carrier protein), a transporter in the inner mitochondrial membrane, transports free carnitine out of the mitochondrial matrix and into the cytoplasm as well as acyl-carnitine into the mitochondrial matrix (Indiveri et al., 2011). Acyl-carnitine entering the mitochondrial matrix is again converted to acyl-CoA by CPT2 and participates in the true fatty acid β-oxidation cycle (Indiveri et al., 2011).

**FIGURE 1:**
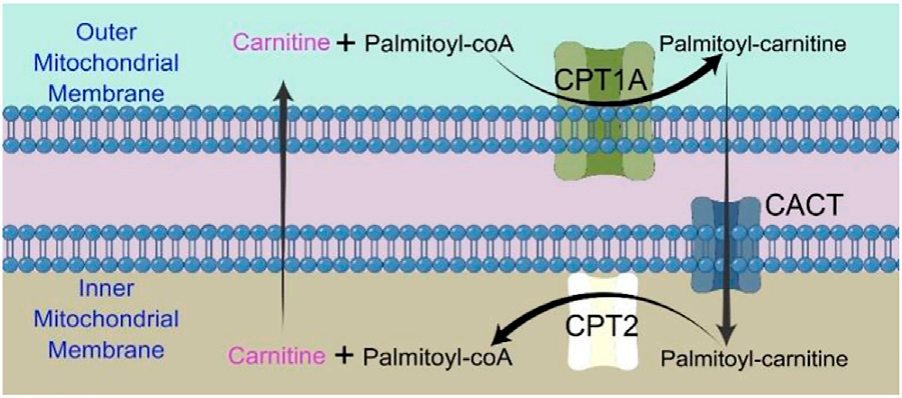
Chart showing how the CPT shuttle system transports palmitoyl-CoA into the mitochondria. The carnitine shuttle system includes CPT1A, CACT, and CPT2. Mitochondrial fatty acid β-oxidation (FAO) is started by the successive actions of CPT1A (in the outer membrane) and CPT2 (in the inner membrane), together with a carnitine-acylcarnitine translocase (CACT).

CPT1A is a rate-limiting enzyme of FAO that catalyzes the transfer of the long-chain acyl group in acyl-CoA ester to carnitine, allowing fatty acids to enter the mitochondrial matrix for oxidation (Figure 1) (Houten et al., 2016).

**FIGURE 2:**
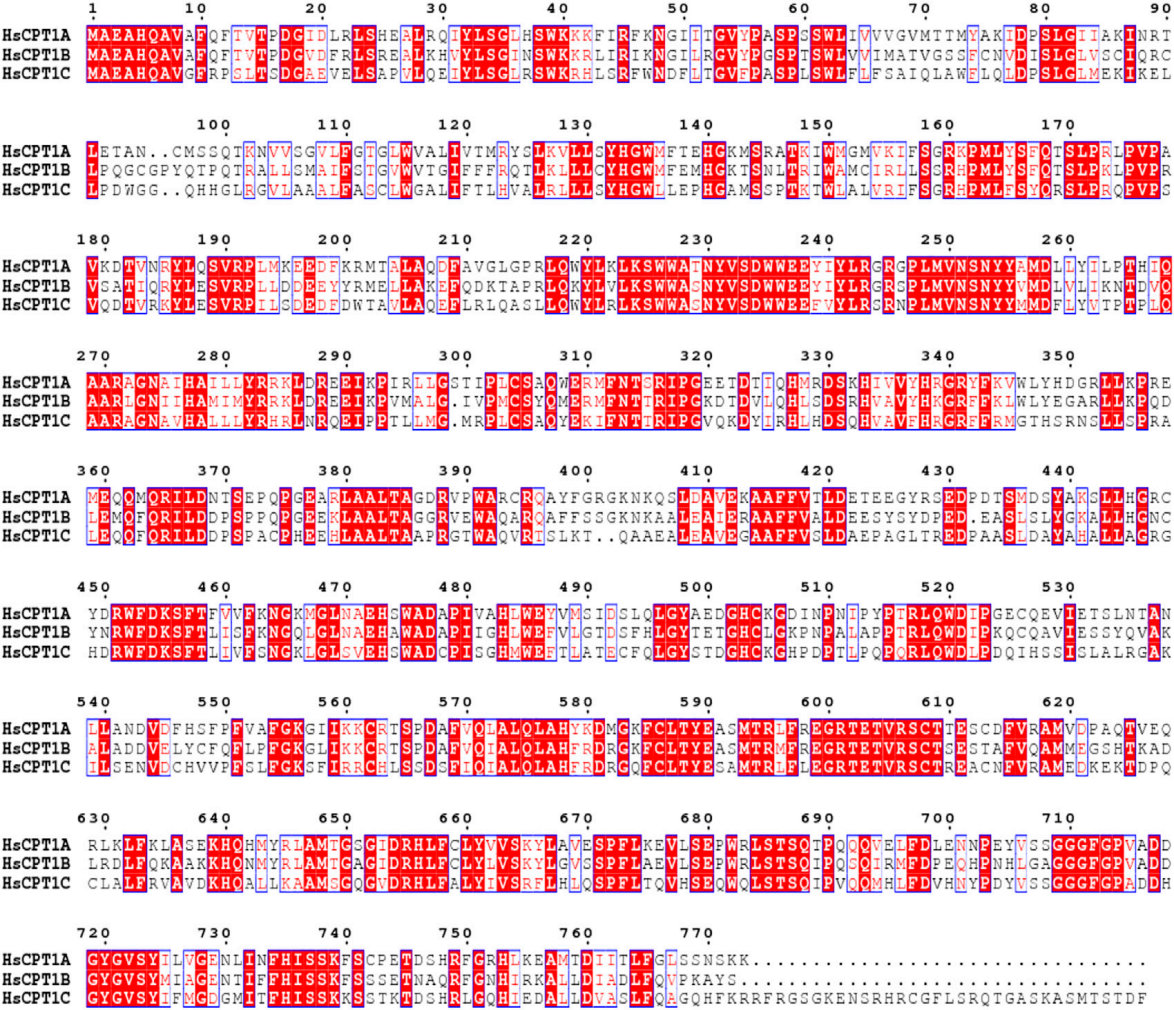
Multiple sequence alignment was performed by ClustalW in MEGA11 software (Tamura et al., 2021) and further treated by ESPript webserver (Robert and Gouet, 2014). Sequence alignment of three CPT1 isoforms with strictly conserved amino acid residues highlighted in box.

CPT1 was originally identified by McGarry and Foster in 1978, and they hypothesized that CPT1 is the rate-limiting enzyme in fatty acid oxidation (McGarry et al., 1978). CPT1A can be divided into three isoforms, known as CPT1A, CPT1B, and CPT1C, based on its tissue distribution, and sequence characteristics. CPT1A is extensively expressed in the liver, kidney, pancreas, adipose tissue, lymphocytes, and fibroblast, while CPT1B and CPT1C have strict tissue-specific distributions (Bonnefont et al., 2004). CPT1A has a higher affinity for carnitine (Km = 30 μM for ratCPT1A) than CPT1B (Km = 500 μM for ratCPT1B) (Ramsay et al., 2001; Ceccarelli et al., 2011). Both CPT1A and CPT1B have significant effects on the metabolic syndrome, cardiovascular disease, type 2 diabetes, and other disorders (Bonnefont et al., 2004). Human CPT1A and CPT1B share 63% of their total sequence homology, 82% around the active region (Figure 2) (Ceccarelli et al., 2011). CPT1C is mostly found in the hypothalamus and hippocampus, where it can control ceramide levels and influence learning, cognition (Virmani et al., 2015). Although CPT1C can bind malonyl-CoA, it has low catalytic activity, making functional investigations difficult (Fado et al., 2021). Spastic paraplegia, which is exclusive to CPT1C and has nothing in common with CPT1A deficiency, is most likely caused by a dominant genetic variant in CPT1C (Rinaldi et al., 2015).

**FIGURE 3:**
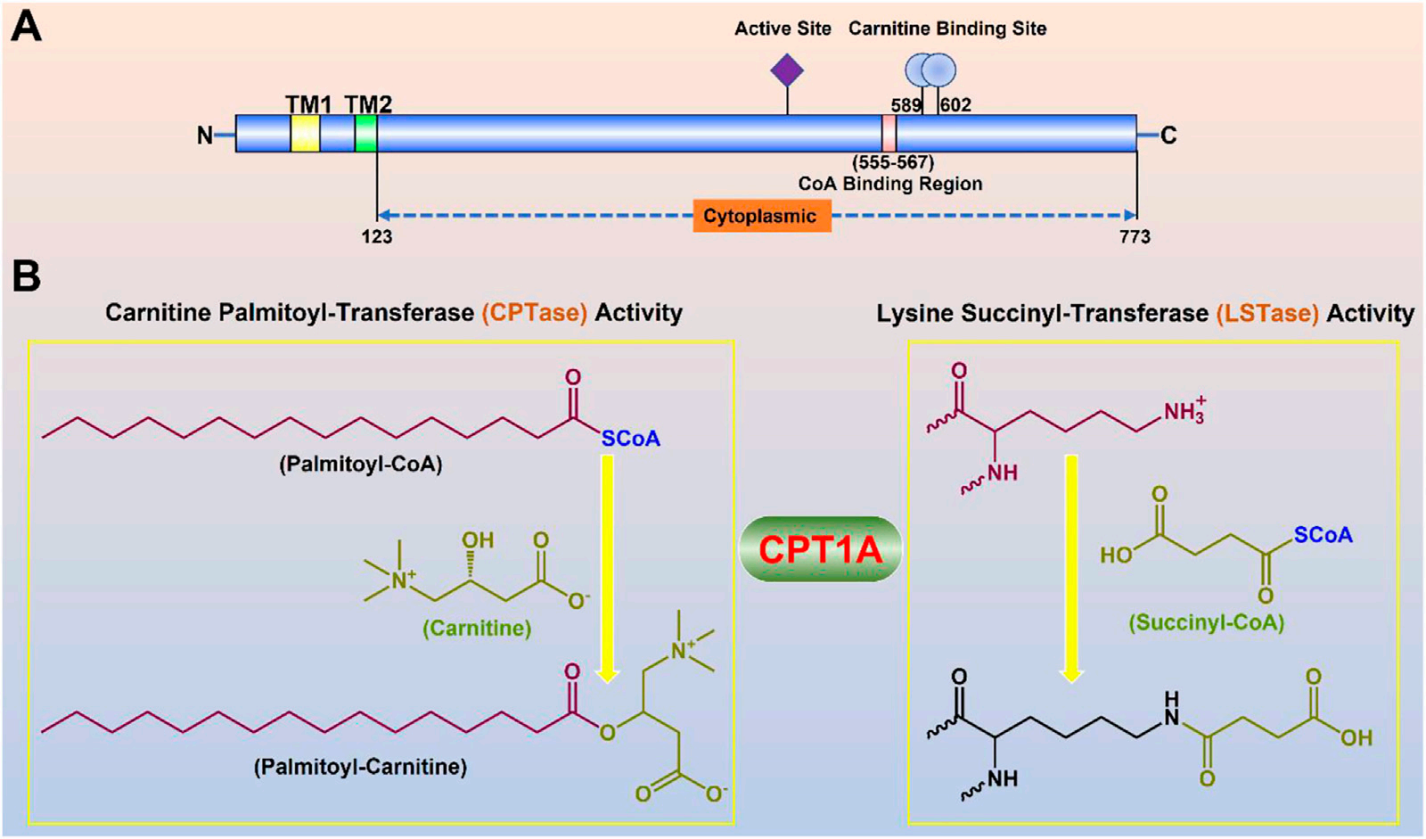
Molecular properties and physiological functions of CPT1A. **(A)** Domain structure of the full-length CPT1A; Two transmembrane helical regions (TM1 and TM2) divide CPT1A protein into the small N-terminal regulatory region (about 47 amnio acids) and the main C-terminal catalytic domain. **(B)** Dual physiological functions of CPT1A.

CPT1A gene encodes a protein with 773 amino acids, which is a transmembrane protein located in the outer membrane of mitochondria facing the cytoplasm (Ramsay et al., 2001). Two transmembrane helical regions divide CPT1A protein into the small N-terminal regulatory region (about 47 amnio acids) and the main C-terminal catalytic domain (Gobin et al., 2003). Near the N-terminal region of CPT1A, two transmembrane helices act as an anchor to the mitochondrial outer membrane. The majority of the N- and C-termini are located on the cytoplasmic side, leaving just a loop of about 27 amino acid residues at the N-terminus in the inner and outer membrane space (Figure 3) (Fraser et al., 1997).

The basic state of CPT1A is a trimer, which has the potential to further merge into a hexamer, and may play a role in the sensitivity to malonyl-CoA (Faye et al., 2007). CPT1A, together with acyl-CoA synthetase (ACSL) and voltage-dependent anion channel (VDAC) form the fatty acid transfer complex located in the outer membrane (Lee et al., 2011).

So far, it is difficult to find a membrane (or membrane-like) condition that can maintain the enzyme activity of CPT1 to make the protein soluble, let alone to study the structure of this enzyme. Despite characterization of the ratCPT2 crystal structure, the sequence similarity with CPT1A is low, making it of little use as a reference for CPT1A structure research (Gobin et al., 2003).

## Structure of CPT1A

CPT1A was discovered in 1978 as the result of a gene located at 11q13.3 that comprises 22 exons and belongs to the carnitine/choline acetyltransferase family (McGarry et al., 1978). The CPT1A gene codes for a 773 amino acid protein that has a short N-terminal regulatory domain (residues 1–47), a mitochondrial intermembrane domain (residues 74–102), two transmembrane (TM) domains (residues 48–73 for TM1 and residues 103–122 for TM2), and a catalytic domain (residues 123–773). A conformation shift in the N-terminal region is critical in malonyl-CoA-mediated enzyme activity control (Rao et al., 2011).

**FIGURE 4:**
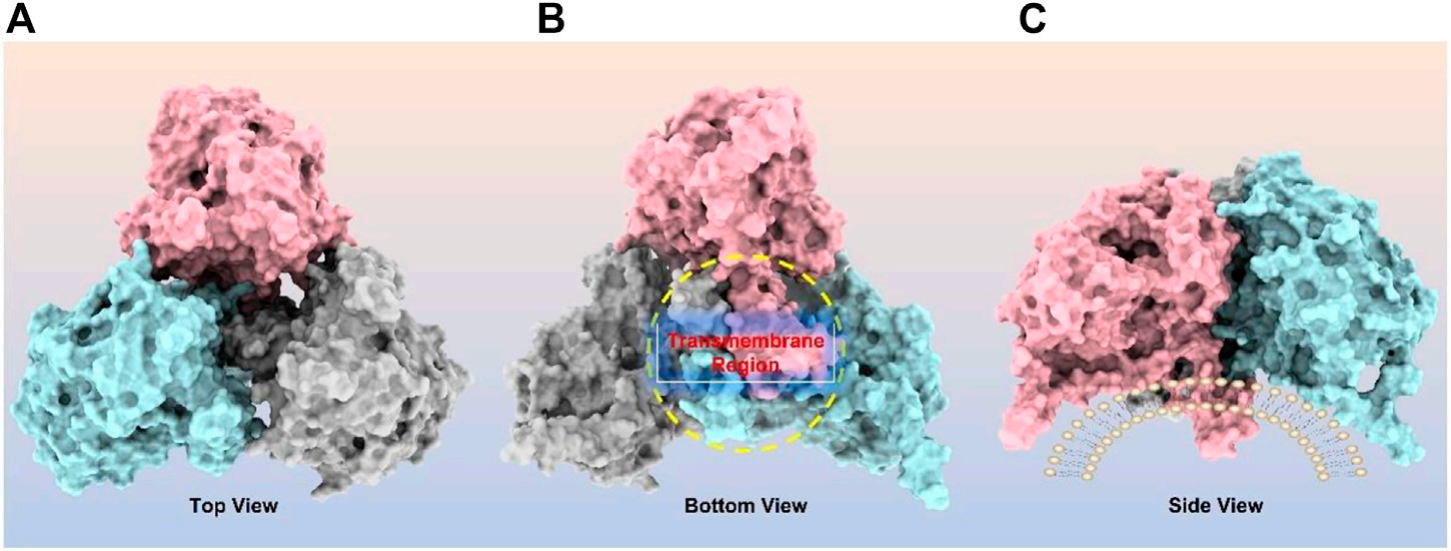
CPT1A homo-trimer structure predicted by ClusPro online server (https://cluspro.org/). **(A)** Top view of CPT1A trimer; **(B)** Bottom view of CPT1A trimer with transmembrane region highlighted by yellow circle; **(C)** Side view of CPT1A trimer located on mitochondrial outer membrane.

CPT1A can be homo-oligomerized to form trimers (Figure 4), which further form hexamers (Faye et al., 2007; Jenei et al., 2009). It has been suggested that the interaction between the GXXXG and GXXXA motifs in CPT1A TM2 helix is essential for its oligomerization (Jenei et al., 2009). Another study revealed that long chain acyl-CoA synthetase 1 (ACSL1) and the voltage-dependent anion channel (VDAC) were also immunocaptured by CPT1A antibodies, suggesting that CPT1A, ACSL1, and VDAC may all be members of mitochondrial outer membrane acylcarnitine translocation complex (Lee et al., 2011). Regarding the oligomerization of CPT1A, both models have a justification for existing due to the lack of structural support, and further (*in situ*) structural studies are needed to resolve this issue.

## Physiological functions of CPT1A

### Succinylation

Lysine succinylation is a newly identified protein post-translational modification (Zhang et al., 2011). CPT1A is one of four well-known succinylation regulators including CPT1A, lysine acetyltransferase 2A (KAT2A), Sirtuin5 (SIRT5) and Sirtuin7 (SIRT7) (Wang et al., 2017; Kurmi et al., 2018; Lu et al., 2021). CPT1A mutant H473A (key acyl-CoA binding site) loses all acylation activity; mutant G710E loses the carnitine palmitoyl-transferase activity, however, maintains its succinylation activity, suggesting that Gly710 is very important for CPT1A succinyl-transferase activity (Kurmi et al., 2018). CPT1A can modulate the succinylation of enolase 1 to enhance the growth of breast cancer (Kurmi et al., 2018) and the succinylation of S100A10 to facilitate the spread of gastric cancer (Wang et al., 2019).

### CPT1A in iTreg cell differentiation

Inducible regulatory T (iTreg) cells are critical for immune suppression and maintains the immune homeostasis (Josefowicz et al., 2012; Savage et al., 2013). Butyric acid can be processed to butyryl-CoA, which competes with malonyl-CoA at His473 to release CPT1A activity for FAO, thereby inducing inducible regulatory T cell (iTreg cell) differentiation *via* Butyric acid- Butyryl CoA—CPT1A axis (Hao et al., 2021).

### Regulation of CPT1A

**FIGURE 5:**
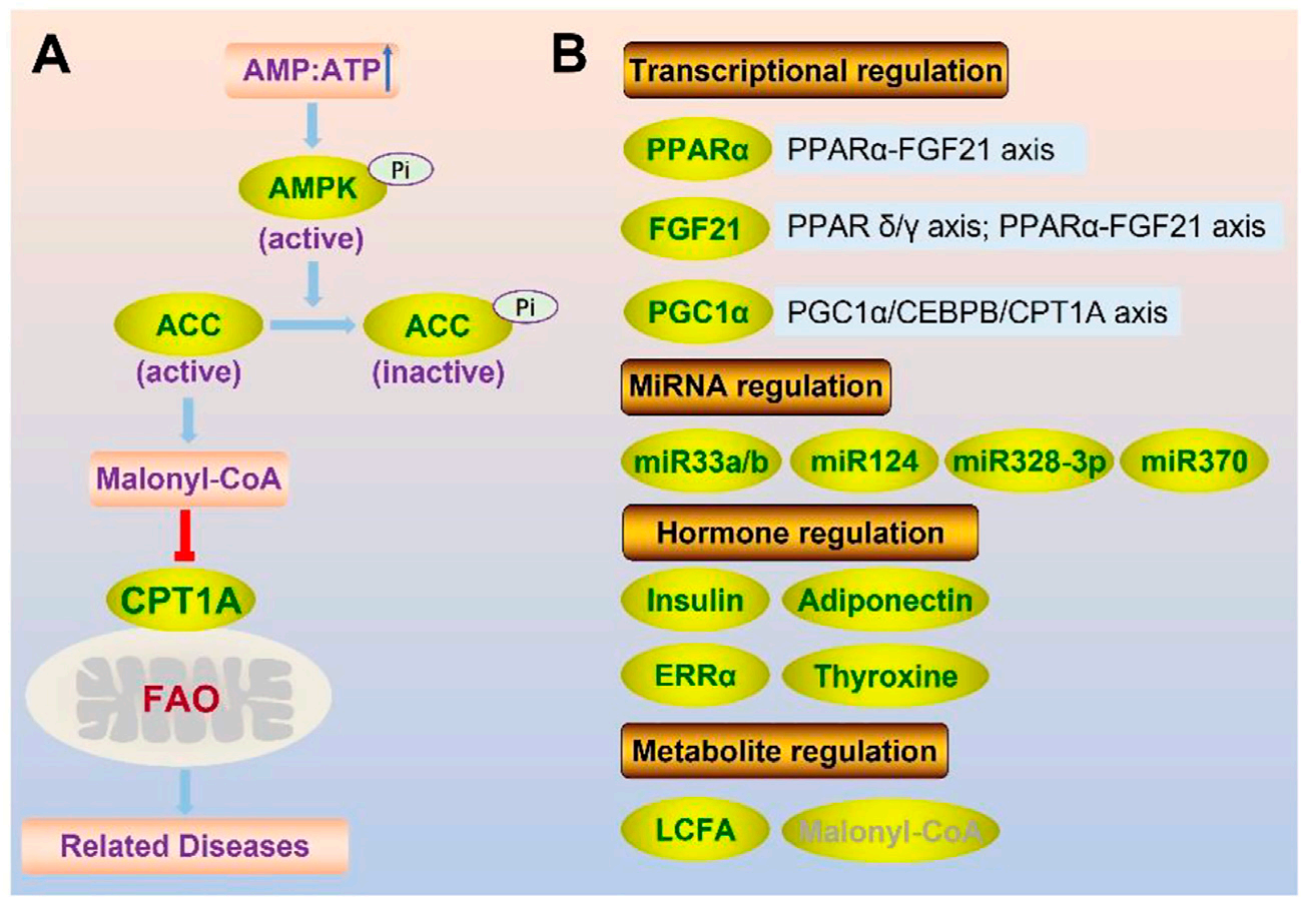
Regulation of CPT1A at protein level (enzyme activity) **(A)** and gene expression level **(B)**.

The balance of lipid metabolism is crucial for maintaining homeostasis, making it crucial to control CPT1A, the key enzyme of FAO. Genetic, physiological, and dietary modulators are all involved in the regulation of CPT1A (Figure 5).

### Transcriptional regulation of CPT1A

*PPARα* Proliferative activated receptor α (PPARα) can greatly enhance CPT1A expression (Kersten et al., 1999). The transcriptional coactivator PPAR gamma coactivator 1α (PGC-1α) cooperates with PPARα to modulate CPT1A in the liver (Jackson-Hayes et al., 2003). Besides, the regulation of CPT1A by Leucine-rich repeat kinase 2 (LRRK2) may be through the activation of AMP-activated protein kinase (AMPK) and PPARα (Lin et al., 2020).

*FGF21* The PPARα-fibroblast growth factor 21 (FGF21) axis was activated in the liver of CPT1A deficient mice, which could be used to avoid inflammation, insulin resistance, and weight gain (Sun et al., 2021). Besides, FGF21 could promote CPT1A expression and FAO in β-cells by activating the AMPK-ACC (acetyl-CoA carboxylase) pathway and PPAR δ/γ signaling axis (Xie et al., 2019).

*PGC-1α* PGC1α binds to CCAAT/enhancer binding protein β (CEBPB) to enhance CPT1A transcription, resulting in activation of FAO through PGC1α/CEBPB/CPT1A/FAO signaling axis, which can promote radiation resistance of nasopharyngeal carcinoma (NPC) (Du et al., 2019).

### MiRNA regulation of CPT1A

*MiR-33a/b* Several genes involved in fatty acid metabolism including CPT1A, HADHB contain predicted binding sites for miR-33a/b (Davalos et al., 2011). Overexpression of miR-33a/b can reduce FAO by downregulating CPT1A and lead to the accumulation of triglycerides in human hepatic cells (Davalos et al., 2011).

*MiR-124* Downregulation of CPT1A expression by miR-124, limiting the conversion of long-chain acyl-CoA moieties to long-chain acylcarnitine (Valentino et al., 2017).

*MiR-328-3p* CPT1A is a downstream target of miR-328-3p in breast cancer, and miR-328-3p overexpression suppresses cancer spread by interfering with FAO *via* CPT1A (Zeng et al., 2022). The MiR-328-3p-CPT1A-FAO pathway is crucial for the metastasis of breast cancer, and miR-328-3p upregulation can be used for reducing metastasis in breast cancer patients (Zeng et al., 2022).

*MiR-370* The liver miR-370 plays a significant role in the inhibition of CPT1A gene expression, which can directly affect its target gene CPT1A, suppressing its expression and lowering FAO efficacy (Iliopoulos et al., 2010).

### Hormone regulation of CPT1A

Hormones can affect CPT1A in organs like the heart, liver, and muscle. Several hormones can take involvement in CPT1A expression and enzyme activity control.

*Insulin* Insulin can significantly lower the expression of CPT1A and increase its sensitivity to malonyl-coA (Park et al., 1995). By controlling CPT1A expression and enzyme activity, insulin can limit FAO and gluconeogenesis in order to lower blood sugar level, which is also one of the mechanisms to treat diabetes.

*Thyroxine* Thyroxine, a different hormonal modulator, has a significant impact on FAO in the liver. CPT1A mRNA could rise by five times with thyroxine supplementation, but mRNA in hypothyroid rats decreased (Heimberg et al., 1985).Thyroxine can increase CPT1A expression by interacting with the thyroid response elements (TRE) in the CPT1A promoter, (Mynatt et al., 1994). Thyroxine can also assist in the upregulation of the CPT1A gene by increasing PGC-1α mRNA and protein levels in hepatocytes (Zhang et al., 2004).

*ERRα* Estrogen-Related Receptor-α (ERRα), which belongs to the nuclear receptor subfamily, is a viable target for NAFLD and that the ERRα agonist may serve as an intriguing pharmacological option for management of metabolic diseases (Mao et al., 2022). ERRα reduces the thyroid hormone-induced expression of CPT1A and mitochondrial FAO *via* PGC1α (Singh et al., 2018). Inhibition of ERRα with XCT790 treatment can increase the expression of CPT1A, further promoting lipid metabolism (Michalek et al., 2011).

*Adiponectin* Adiponectin, an adipose-secreted protein that has been linked to insulin sensitivity, plasma lipids, and inflammatory patterns, is an established biomarker for metabolic health (Aslibekyan et al., 2017). Adiponectin phosphorylates and triggers AMPK, which modulates CPT1A *via* the AMPK-ACC-CPT1A pathway (Li et al., 2007). The acetyl-CoA carboxylase (ACC)/malonyl-CoA pathway could be strongly blocked by phosphorylated AMPK, thus increasing the activity of the CPT1A enzyme. Besides, CPT1A methylation is associated with circulating adiponectin levels, likely in an obesity-dependent manner, which can be a novel pleiotropic marker of chronic disease risk (Aslibekyan et al., 2017).

### Metabolite regulation of CPT1A

*Long chain fatty acid (LCFA)* LCFA is a substrate for CPT1A and one natural ligand of PPARα (Nakamura et al., 2014). It can up-regulate CPT1A by directly acting on peroxisome proliferator response elements (PPREs) in CPT1A introns, as well as *via* activating PPARα (Chatelain et al., 1996; Le May et al., 2005).

*Malonyl-CoA* Malonyl-CoA is produced by acetyl-CoA carboxylase2 (ACC2) during fatty acid synthesis, and is a natural inhibitor of CPT1A. CPT1A is sensitive to malonyl-CoA, and the sensitivity depends on the concentration of malonyl-CoA (Robinson and Zammit, 1982). However, fasting or insulin deficit significantly reduces CPT1A’s sensitivity to malonyl-CoA (Park et al., 1995; Akkaoui et al., 2009). CPT1A structure has a short N-terminus and a major C-terminus containing a catalytic site and a malonyl-CoA binding site. Malonyl-CoA inhibition of CPT1A will be lost as a result of the specific area of the N-terminal interacting with the malonyl-CoA binding site in the C-terminal (Morillas et al., 2004; Lopez-Vinas et al., 2007; Rufer et al., 2009; Rao et al., 2011).

Malonyl-CoA inhibits CPT1A activity through allosteric inhibition and in a concentration-dependent manner, and the C-termini of CPT1A served as the malonyl-CoA binding site (Rao et al., 2011). The affinity for malonyl-CoA can also be influenced by the N-terminal state of CPT1A (residues 1–42), which has two possible conformational states (Nα inhibitory state and Nβ non-inhibitory state) (Lopez-Vinas et al., 2007; Rao et al., 2011). The presence of a curved, amphiphilic binding surface is necessary for the non-inhibitory state (Rao et al., 2011).

**FIGURE 6:**
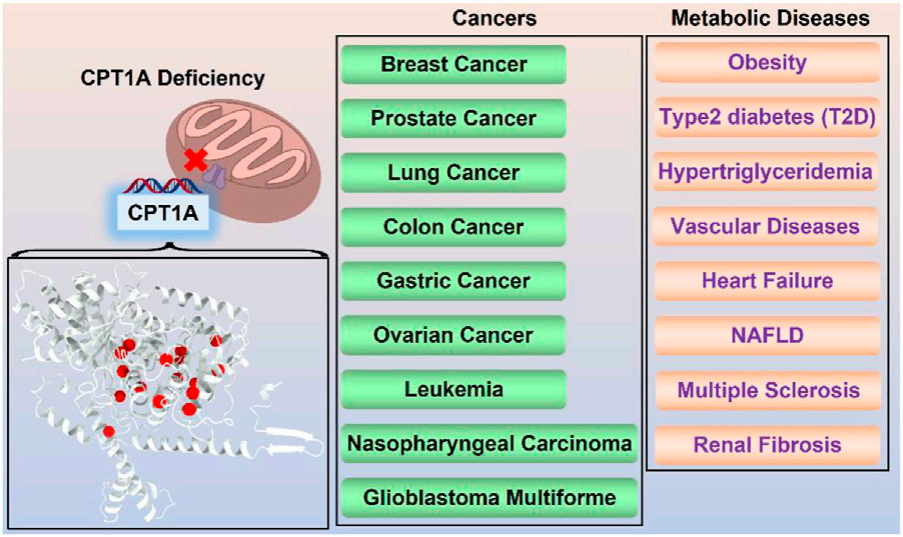
Summary of CPT1A-related diseases.

CPT1A activity can be regulated *via* the AMPK-ACC-CPT1A axis (Figure 6) (Yao et al., 2020). ACC is inhibited through phosphorylation by AMPK, and ACC’s inhibiting effect on CPT1A is relieved by lowering the malonyl-CoA level (Yao et al., 2020).

## CPT1A in diseases

### CPT1A deficiency

**FIGURE 7:**
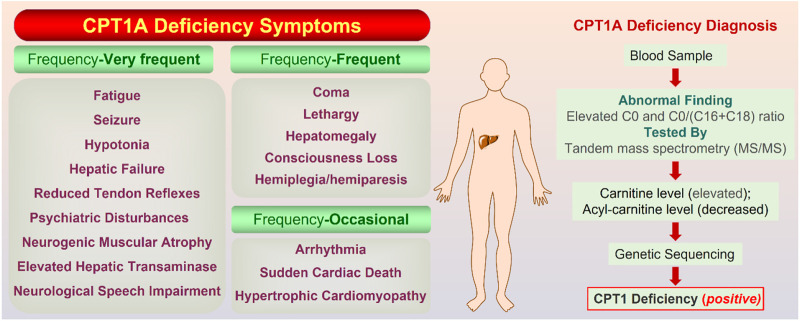
On the left are the symptoms of CPT1A deficiency gathered from Genetic and Rare Diseases (GARD) Information Center (https://rarediseases.info.nih.gov/). On the right is the confirmatory screening process for CPT1A deficiency diagnosis. The normal value of the C0/(C16 + C18) ratio is 278.75; Total carnitine is normal at 33–70 mmol/L and free carnitine is normal at 28–52 mmol/L (Dowsett et al., 2017).

CPT1A deficiency is a rare mitochondrial FAO disorder caused by autosomal recessive mutations (Bellusci et al., 2017). The CPT1A deficiency can trigger a variety of illnesses, including hepatic encephalopathy, recurrent hypoglycemia, hepatomegaly, hyperammonemia, renal tubular acidosis, and so on (Collins et al., 2010). Rapid onset, frequent recurrence, and high mortality are significant features for CPT1A deficiency. Symptoms are classified into three classes according to the frequency (Figure 7).

Mutations associated with CPT1A deficiency can be divided into two categories: type one affect directly the catalytic center, which results in loss of activity (functional determinant); type two affects the stability of the enzyme, which indirectly decreases the catalytic efficiency (structural determinant) (Gobin et al., 2003).

**FIGURE 8:**
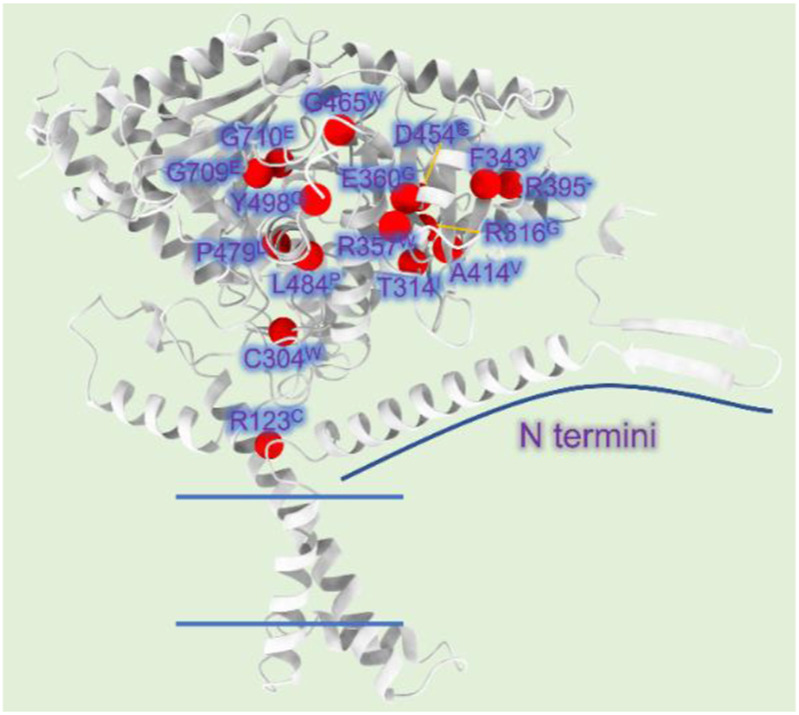
CPT1A deficiency related mutations reported in patients. (A) The CPT1A structure (from AlphaFold database) is colored by grey. The Cα atoms of the CPT1A mutations in patients are shown as red spheres. R123C, C304W, T314I, R316G, F343V, R357W, E360G, R395 (missing), A414V, D454G, G465W, P479L, L484P, Y498C, G709E, and G710E are among the missense mutations discovered in CPT1A.

Mutations A275T, R357W, A414V, L484P, and Y498C, far away from the active region, decrease activity by affecting the stability of CPT1A (Gobin et al., 2003). C304W, P479L, and R395 deletion almost lose activity (Brown et al., 2001). High mortality rate mutants P479L is a frequent CPT1A mutation found in Arctic regions like Canada (Rajakumar et al., 2009; Gessner et al., 2010). Additionally, G709E and G710E, two sites essential for the hydrophobic core in the catalytic site, can abolish CPT1A activity (Dai et al., 2000). The hydrophobic catalytic core is altered when Gly709 or Gly710 is changed to a Glu, which introduces a large and negatively charged group (Gobin et al., 2003). This mutation is close to the catalytic His473 residue and the carnitine binding site, which can significantly change the hydrophobic pocket of CPT1A (Gobin et al., 2003). We compiled the majority of the reported natural mutations so far and mapped them onto CPT1A structure model (Figure 8) (Table 1).

**TABLE 1:** Natural mutations of CPT1A found in patients.

Gene	Protein	Disease Description	Effect	References
c.367C>T	R123C	CPT1A deficiency	—	Brown et al. (2001)
c.912C>T/c.912C>G	C304W	CPT1A deficiency	decreased stability	Brown et al. (2001)
c.941C>T	T314I	CPT1A deficiency	—	Stoler et al. (2004)
c.946C>G	R316G	CPT1A deficiency	—	Bennett et al. (2004)
c.1027T>G	F343V	CPT1A deficiency	—	Bennett et al. (2004)
c.1069C>T	R357W	CPT1A deficiency	decreased stability	Brown et al. (2001)
c.1079A>G	E360G	CPT1A deficiency	reduced protein levels	Ogawa et al. (2002)
—	R395 (missing)	CPT1A deficiency	loss of activity	Brown et al. (2001)
c.1241C>T	A414V	CPT1A deficiency	decreased activity	Gobin et al. (2002), Gobin et al. (2003)
c.1361A>G	D454G	CPT1A deficiency	loss of activity	IJlst et al. (1998)
c.1393G>T	G465W	CPT1A deficiency	—	Bennett et al. (2004)
c.1436C>T	P479L	CPT1A deficiency	decreased activity	Brown et al. (2001)
c.1451T>C	L484P	CPT1A deficiency	decreased stability	Brown et al. (2001)
c.1493A>G	Y498C	CPT1A deficiency	decreased activity	Gobin et al. (2002), Gobin et al. (2003)
c.2126G>A	G709E	CPT1A deficiency	loss of activity	Gobin et al. (2003)
c.2129G>A	G710E	CPT1A deficiency	loss of activity	Prip-Buus et al. (2001), Gobin et al. (2003)

Currently, there is no specific drug for CPT1A deficiency, and diet control is the main therapy. Tubular acidosis can be improved after treatment with medium chain triglyceride in CPT1A deficiency patients (Falik-Borenstein et al., 1992). Therefore, timely screening and diagnosis of CPT1A defects appear to be particularly important, and CPT1A defects can be finally confirmed based on results from multiple aspects (Figure 7).

### CPT1A and metabolic diseases

Patients with metabolic syndrome, obesity, and type 2 diabetes frequently have non-alcoholic fatty liver disease, hypertriglyceridemia, and other lipid metabolism abnormalities, which can be somewhat improved by increasing CPT1A expression (Deprince et al., 2020). Increased CPT1A expression has been shown to drastically lower liver triglyceride levels (Stefanovic-Racic et al., 2008), and suppress JNK factor to prevent the inflammatory response brought on by free fatty acids (Gao et al., 2011). Fatty acid accumulation can cause the development of insulin resistance, which can eventually result in type 2 diabetes and hyperinsulinemia (Levin et al., 2007; Pan et al., 1997).

#### CPT1A in vascular diseases

Metabolism of endothelial cell depends on FAO to resist oxidative stress in the development of blood vessels, and CPT1A plays a crucial role in this process, offering a new potential target for the treatment of vascular-related diseases (Rohlenova et al., 2018).

#### CPT1A in heart failure

Heart failure is one of the top causes of death and disability around the world, but there is yet no safe and effective clinical therapy for heart failure (Bui et al., 2011). The clinical study of the CPT1A inhibitor etomoxir showed improvement in heart failure, but was ended prematurely due to elevated liver transaminase in enrolled patients (Holubarsch et al., 2007).

Perhexiline was an effective anti-angina drug used in the last century, but was recalled by the manufacturer because of hepatotoxicity and peripheral neurotoxicity (Ashrafian et al., 2007). Later studies confirmed that Perhexiline could selectively block CPT1A in liver and heart, and had an ideal inhibitory effect on FAO and improved the oxidation of carbohydrate in the heart (Ashrafian et al., 2007).

#### CPT1A in NAFLD

The global obesity epidemic has dramatically increased the prevalence of non-alcoholic fatty liver disease (NAFLD), with no approved treatment now (Weber et al., 2020). Deficient CPT1A expression in the liver results in a healthy steatotic state that protects against high-fat diet-induced liver damage and increases adipose browning in a PPARα-FGF21 axis dependent manner, suggesting that inhibition of hepatic CPT1A may serve as a viable strategy for the treatment of obesity and NAFLD (Weber et al., 2020; Sun et al., 2021).

#### CPT1A in multiple sclerosis

Inuits have a low prevalence of multiple sclerosis, possibly associated with in CPT1A P479L mutation (Morkholt et al., 2019). This point mutation result in 22% residual activity of the CPT1A (Collins et al., 2010). CPT1A inhibition may represent a prospective therapeutic therapy for multiple sclerosis.

#### CPT1A in renal fibrosis

Renal fibrosis is a result of several types of chronic kidney diseases, and currently, the only treatment is to control blood pressure and blood sugar levels (Drawz and Rosenberg, 2013). With conditional overexpression of CPT1A, mitochondrial dysfunction in the fibrosis kidney can be alleviated, and renal fibrosis can be significantly decreased (Miguel et al., 2021). Gain of function in CPT1A strategy may be a novel approach to treating fibrosis in renal fibrosis (Miguel et al., 2021).

### CPT1A and cancer

Oxidative stress is the key element that causes prostate cancer to develop (Khandrika et al., 2009). Androgens may raise the levels of CPT1A and the accumulation of reactive oxygen species, which are closely linked to prostate cancer cell proliferation and differentiation (Joshi et al., 2020). Currently, the primary treatments for prostate cancer include diet modification and the use of antioxidants, while CPT1A inhibition may provide novel therapeutic options (Lin et al., 2010).

One characteristic of cancer is metabolic reprogramming, which provides tumor cells the basic elements for fast cell growth and maintains cell survival under hypoxic or nutrient-deficient conditions (Yoshida, 2015). Abnormal fat metabolism has a profound impact on cell carcinogenesis. As an important source of NADH, NADPH, FADH2 and ATP, FAO plays a key role in various stages of tumor occurrence, development and metastasis (Wang et al., 2021). CPT1A is the critical rate-limiting enzyme in FAO, responsible for transporting fatty acids from cytoplasm to mitochondria for oxidation. There is evidence that CPT1A is crucial for metabolic adaptability in the development of cancer, and CPT1A inhibition slows the spread of cancer (Tang et al., 2022).

According to growing amounts of experimental evidence published in reputable journals in recent years, CPT1A may be a significant drug target for a number of cancer cells, including breast cancer (Xiong et al., 2018; Tan et al., 2021), prostate cancer (Schlaepfer et al., 2014; Joshi et al., 2019), glioblastoma (Jiang et al., 2022; Kim et al., 2022; Luo et al., 2022), colon cancer (Wang et al., 2018), gastric cancer (Wang et al., 2019; Wang et al., 2020), multiple myeloma (Shi et al., 2016), nasopharyngeal cancer (Tan et al., 2018; Tang et al., 2022), etc. CPT1 plays an important role in the occurrence and development for these cancers, and pharmacological inhibition of CPT1A can effectively inhibit cancer cell proliferation, which makes CPT1 a possible molecular marker for tumor diagnosis and a new target for anti-tumor therapy.

#### CPT1A in breast cancer

Breast cancer is the most prevalent and leading cause of cancer death among women globally, which has a poor prognosis, a high rate of recurrence and metastasis, and a high fatality rate (Waks and Winer, 2019). In 2020, there were 2.26 million new instances of breast cancer worldwide, overtaking lung cancer (2.21 million) for the first time to take the top place among all cancers, according to the International Agency for Research on Cancer’s (IARC) estimate of the global cancer (Ferlay et al., 2021).

Invasion and lymphangiogenesis in breast cancer cells can be inhibited by CPT1A knockdown, and CPT1A-null Human Dermal Lymphatic Endothelial Cells (HDLEC cells) consistently showed impaired invasion and lymphangiogenesis (Xiong et al., 2018). CPT1A knockdown reduced the expression of lymphangiogenic markers like Vascular endothelial growth factor receptor-3 (VEGFR-3) in HDLEC cells *via* acetyl-CoA-mediated Histone H3 lysine 9 acetylation (H3K9ac), which can be reversed by the addition of acetate (Xiong et al., 2018).

Myc-overexpressing triple-negative breast cancer (TNBC) has a greater bioenergetic dependence on FAO, and CPT1A can be pharmaceutically inhibited to reduce energy metabolism in Myc-overexpressing TNBC cells and stop tumor growth in a xenograft model of Myc-overexpressing TNBC (Camarda et al., 2016; Park et al., 2016). This suggests that CPT1A inhibition may be a promising therapeutic approach for this particular subtype of breast cancer.

Besides, Chemotherapy (CT) and radiotherapy (RT) target rapidly dividing cells but still have significant normal tissue toxicity. One indicator of RT and CT resistant tumor cells is thought to be the abnormal upregulation of CPT1A-dependent FAO (Corn et al., 2020). High CPT1A expression, increased FAO, and a poor prognosis are characteristics of radiation-resistant breast cancer cells (Corn et al., 2020). Radiation resistant breast cancer cells respond to ionizing radiation by increasing FAO and ATP production, resulting in increased phosphorylation of extracellular signal regulated kinase 1/2 (ERK1/2), decreased apoptosis, and promotes a more aggressive phenotype (Han et al., 2019). Drug candidates such as Etomoxir or its analogs, which inhibits CPT1A and FAO, can be developed as RT and CT sensitizers in breast cancer (Han et al., 2019).

#### CPT1A in prostate cancer

The incidence of prostate cancer ranks the second among male malignant tumors in the world (Schlaepfer et al., 2014). In United States, prostate cancer has surpassed lung cancer, becoming the most serious malignant tumor (Carlsson et al., 2012). China has lower rates of prostate cancer than Western nations, but these rates have been rising recently (Culp et al., 2020).

Rather than using aerobic glycolysis, prostate cancer prefers lipid for fuel. Prostate cancer cells may have less vitality after therapy with etomoxir, irreversible inhibitor of CPT1A, and etomoxir treatment in mice reduced xenograft growth for a period of 21 days (Schlaepfer et al., 2014). Reduced mTOR signaling, elevated caspase-3 activation, and decreased androgen receptor expression are linked to these outcomes (Schlaepfer et al., 2014). The growth of prostate cancer may be aided by a stress state caused by reactive oxygen species (ROS) that is linked to CPT1A overexpression (Joshi et al., 2020). Besides, increased histone acetylation has been observed in prostate cancer cells that over-express CPT1A, suggesting that acetylation may be a means by which CPT1A controls prostate cancer cell proliferation (Joshi et al., 2019). These facts highlight the therapeutic potential of CPT1A blockade to prevent prostate cancer.

#### CPT1A in lung cancer

Lung cancer is one of the most common malignant tumors in humans, and its incidence and mortality are increasing year by year worldwide (de Sousa and Carvalho, 2018). Cisplatin is one widely used chemotherapy drugs for lung cancer (O'Byrne et al., 2011; Wang and Lippard, 2005). Knockdown of CPT1A can promote tumor cell susceptibility to Cisplatin. The CPT1A inhibitor etomoxir can affect and coordinate with the conventional chemotherapy drug cisplatin to increase tumor cell sensitivity to the chemotherapeutic agent, inhibit tumor cell proliferation and promote apoptosis, thus providing a novel approach to improving the efficacy of chemotherapy in non-small cell lung cancer (Dheeraj et al., 2018; Hoy et al., 2021).

#### CPT1A in glioblastoma multiforme (GBM)

GBM is the most common and difficult central nervous system malignancies, with a 5-year survival rate of 6.8% (Ostrom et al., 2020). Radiotherapy is the primary treatment for GBM, and radiotherapy plus immunotherapy is emerging as a new option due to the strong resistance and poor efficacy of GBM to radiotherapy (Arina et al., 2020). However, tumor cells after radiotherapy develop a tolerance to immunotherapy, allowing tumor cells to escape the killing of immune cells, leading to treatment failure (Darragh et al., 2018). After treatment of radio-resistant GBM cells with the CPT1A inhibitor etomoxir, Oxygen Consumption Rate (OCR) and ATP production were significantly inhibited, suggesting that energy conversion from glycolysis to FAO occurs in radio-resistant GBM cells (Jiang et al., 2022). CPT1A and CD47 are highly expressed in radiotherapy resistant GBM tumors, and inhibition of CPT1A can result in decreased CD47 expression and increased macrophage phagocytosis of tumor cells (Jiang et al., 2022). High expression of CPT1A not only enhances radiotherapy resistance in GBM tumor cells, but also enhances immune escape of macrophages through CD47, suggesting CPT1A as a novel strategy for the treatment of recurrent GBM multiforme.

#### CPT1A in colon cancer

Colon cancer develops in adipose-rich microenvironment, and CPT1A overexpression is crucial for adipocytes to promote tumor growth in colon cancer (Wen et al., 2017; Pearce et al., 2018). CPT1A upregulation is a key metabolic alteration that cancer cells use to promote β-catenin acetylation and activation, while knockdown of CPT1A can reduce the expression of genes linked with colon cancer cells downstream of Wnt/β-catenin (Wen et al., 2017). Overall, CPT1A inhibition may be a useful strategy to lessen the promotion impact of adipocytes on colon cancer.

#### CPT1A in gastric cancer

Gastric cancer is a malignancy of stomach lining (Torre et al., 2015; Sung et al., 2021). In gastric cancer, the calcium-binding cytosolic protein S100A10 is overexpressed and is essential for the invasion and migration of tumor cells (El-Rifai et al., 2002). CPT1A succinylated S100A10 at lysine 47, and the degree of succinylation was elevated in human gastric cancer (Wang et al., 2019). In summary, S100A10 succinylation promotes gastric cancer progression and is regulated by CPT1A-mediated succinylation and sirtuin5 (SIRT5)-mediated desuccinylation (Wang et al., 2019).

#### CPT1A in ovarian cancer

CPT1A was found to be highly expressed in ovarian cancer, and its overexpression is linked to a poor survival in ovarian cancer patients (Shao et al., 2016; Tan et al., 2018). CPT1A inactivation reduced cellular ATP levels and caused cell cycle arrest at G0/G1 stage, implying that ovarian cancer cells rely on CPT1A-mediated FAO for cell cycle progression (Shao et al., 2016).

#### CPT1A in nasopharyngeal carcinoma

Nasopharyngeal carcinoma (NPC) incidence can be affected by genetic susceptibility, and environmental factors. In NPC cells, CPT1A was the only up-expressed carnitine palmitoyl transferase (Tang et al., 2022). Upregulated CPT1A enhances the production of nucleoside metabolic intermediates that promote cell cycle progression is increased in NPC cells (Tang et al., 2022). Belgian scientists revealed *via* isotope labeling that palmitate-derived carbons considerably augmented the Krebs cycle and could be integrated into nucleotide precursors such as aspartic acid, and pyrimidine nucleoside triphosphate (Schoors et al., 2015). Inhibiting CPT1A causes cells to deplete stored aspartic acid and dNTP, impairs *de novo* dNTP synthesis, and inhibits NPC cell cycle’s DNA replication at G1/S transition, implying a potential treatment strategy for NPC based on lipid metabolism regulation (Schoors et al., 2015; Tan et al., 2018).

Furthermore, radiation resistance is still a significant barrier for NPC treatment (Chua et al., 2016; Tan et al., 2018). NPC radiation resistance may be enhanced *via* the PPAR coactivator-1α (PGC1α)/CCAAT/enhancer binding protein β (CEBPB)/CPT1A/FAO signaling axis (Du et al., 2019). Radiation-resistant NPC cells consistently displayed active up-regulation of CPT1A, and inhibition of CPT1A could render NPC cells once again vulnerable to radiation treatment by inducing mitochondrial apoptosis (Tan et al., 2018).

#### CPT1A in leukemia

In acute myeloid leukemia (AML), overexpression of CPT1A indicates poor clinical prognosis, and strong synergistic inhibitory effects on AML were seen when the CPT1A-selective inhibitor ST1326 and the Bcl-2 inhibitor ABT199 were applied in combination (Mao et al., 2021). Overall, CPT1A expression is abnormally high in AML, and targeted suppression of CPT1A has potent anti-leukemic effects, suggesting that CPT1A might be a therapeutic target for the treatment of AML (Mao et al., 2021).

#### CPT1A as a target for cancer treatment

CPT1A inhibitors can lessen the survivability of cancer cells, so CPT1A may be a useful target for cancer therapy. The main drawback of CPT1A blockage is the undesirable impact on non-tumor cells given the extensive tissue distribution of CPT1A. Unfortunately, there has not yet been any evidence of apparent selectivity against other CPT1 isoforms when developing small molecules as CPT1A inhibitors.

## CPT1A and drug development

CPT1A is an intriguing target with significant potential for pharmacological application. For decades, drugs targeting CPT1A have been the focus of research on diseases like type 2 diabetes (T2D), obesity, and other disorders (Rufer et al., 2009). Therefore, drug development targeting CPT1A has attracted much attention.

### CPT1A inhibitors

**FIGURE 9:**
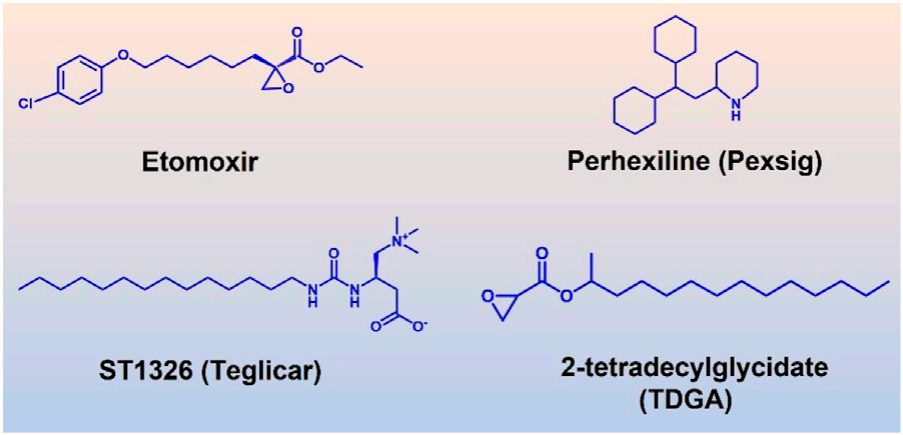
Four well-studied inhibitors (Etomoxir, Perhexiline, ST1326, TDGA) of CPT1A.

The efforts to study the molecular mechanisms of CPT1A inhibition in disease intervention have increased in recent years due to the association with cancer (Schlaepfer et al., 2014). However, there are few small-molecule inhibitors of CPT1A. In the past, efforts were concentrated on CPT1A inhibitors, primarily two main kinds of inhibitors including substrate derivatives, glycidic acid derivatives, and malonyl-CoA analogues (Figure 9) (Ceccarelli et al., 2011).

Etomoxir and 2-tetradecylglycidic acid (TDGA) are both ethylene oxide compounds. Etomoxir and TDGA can bind to the active site of CPT1A to produce inhibitory effects (Brady and Brady, 1986; Ratheiser et al., 1991). These two inhibitors belong to the first developed class. However, Etomoxir lacks selectivity to CPT1 isoforms, and TDGA can affect the renin angiotensin system, resulting in myocardial hypertrophy and other side effects (Brady and Brady, 1986). Etomoxir is a strong irreversible inhibitor of CPT1A (Selby and Sherratt, 1989). However, preclinical research was stopped because of hepatotoxicity after a phase II clinical trial (Holubarsch et al., 2007).

An amino-carnitine analog called ST1326 (oral formulation-Teglicar) was initially created for diabetic ketoacidosis (Giannessi et al., 2003). ST1326 belongs to formyl-carnitine derivatives, which has a specific and reversible inhibitory effect on liver CPT1A, and is currently used as a novel anti-hyperglycemic drug (Conti et al., 2011). Compared to etomoxir, ST1326 (oral formulation-Teglicar) is more selective for CPT1A while CPT1B can also be inhibited by etomoxir (Conti et al., 2011). ST1326 can significantly improve hyperglycemia and adjust the dynamic balance of glucose in obesity and type 2 diabetes models, showing good application prospects (Conti et al., 2011).

It was discovered that C75 directly stimulated CPT1A activity (Idrovo et al., 2012). However, it was also found that CPT1A is inhibited by low amounts of C75 that have been converted to C75-CoA (Mera et al., 2009). There is still debate about this matter, though.

Perhexiline, a clinical CPT1A/CPT2 inhibitor, is approved for the management of angina pectoris outside of the United States (Ashrafian et al., 2007). Perhexiline increases the efficiency of oxygen consumption by blocking FAO and switching the energy metabolism from lipid to carbohydrate. Despite being effective for angina pectoris, perhexiline was reported with neurotoxicity and hepatotoxicity for prolonged use (Ren et al., 2020).

CPT1A is a significant therapeutic target due to its important function in a variety of illnesses (Schlaepfer and Joshi, 2020). CPT1A’s protein structure is still unknown, though. It is crucial to discover the structure and catalytic role of the enzyme in order to design more powerful inhibitors. To avoid side effects, selectivity needs to be considered further. For instance, the fact that perhexiline targets both CPT1A and CPT2 raises the possibility that the negative effects may be related to its lack of isoform selectivity.

The isoform selectivity of CPT1A inhibitor has a significant impact on how the therapeutic benefit is evaluated. The best-studied CPT1 inhibitor, etomoxir, may have the problem of off-target side effect (Yao et al., 2018). When assessing safety, liver selectivity should also be taken into account for CPT1A.

### CPT1A agonists

**FIGURE 10:**
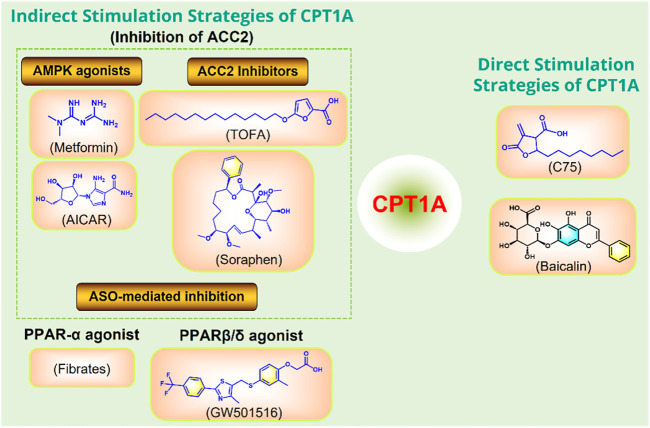
Strategies to directly or indirectly stimulate CPT1A. ASO, antisense oligonucleotide; PPAR, peroxisome proliferator activated receptor; ACC, acetyl-CoA carboxylase; AMPK, adenosine monophosphate-activated protein kinase.

Moderately increasing the expression or activity of CPT1A can promote FAO and improve a variety of metabolic diseases caused by high fat diet (Figure 10).

C75 and baicalin can directly activate CPT1A (Idrovo et al., 2012; Fang et al., 2020). In addition, several small molecules can indirectly activate CPT1A by acting on ACC2, including the AMPK activators metformin and AICAR (Tomita et al., 2005), and the ACC2 inhibitors TOFA and Soraphen (Agostini et al., 2022). Anti-Sense Oligonucleotide (ASO)-mediated inhibition of ACC2 may also play a role in activating CPT1A. CPT1A can also be indirectly promoted by PPAR activators Fibrates and GW501516 (Cox, 2017).

However, overexpression of CPT1A may increase the FAO rate, promote cell metabolism, and accelerate cell apoptosis, which brings certain difficulties for the development of related drugs.

## Conclusion and perspectives

In summary, as a key enzyme in FAO, CPT1A affects the occurrence and development of a variety of diseases. The physiological function and impact of CPT1A are becoming increasingly better understood, which has important guiding significance for the research of CPT1A related diseases, and also provides support for the drug development and application based on targeting CPT1A. CPT1A has become a focus of pharmaceutical research due to CPT1A pathogenic mutations and abnormal expression in malignancies. Interest in CPT1A’s role in cancer has increased recently. The mechanisms through which CPT1A aids in cancer cell survival remain not fully clarified. Given that CPT1A was reported to promote anoikis-resistance and metastasis in cancers like colorectal cancer, CPT1A would be a desirable target to counteract resistance of anticancer drugs (Wang et al., 2018). Although currently available small-molecule drugs targeting CPT1A have shown promising therapeutic effects, their off-target effects and side effects are still the biggest obstacles to their application. Finally, further research on the structure of CPT1A will continue to improve the specificity of drug selectivity to CPT1A to avoid off-target effect and other undesired side effects, which will provide safer and more effective drugs for the clinical therapy.
